# Emerging Role of Transcription Factor 19 (TCF19) in Inflammatory Disease and Cancer

**DOI:** 10.3390/biom16040600

**Published:** 2026-04-17

**Authors:** Xiang Li, Yi-Fang Jiang, Ran Wang, Jing Yu, Yan-Jun Liu, Yun-Fei Dang, Guan-Jun Yang, Jiong Chen

**Affiliations:** 1 State Key Laboratory for Quality and Safety of Agro-Products, Ningbo University, Ningbo 315211, China; 2511130038@nbu.edu.cn (X.L.); 2511130027@nbu.edu.cn (Y.-F.J.); 2411130037@nbu.edu.cn (R.W.); 2411130055@nbu.edu.cn (J.Y.); 22021130072@nbu.edu.cn (Y.-J.L.); dangyunfei@nbu.edu.cn (Y.-F.D.); 2 Laboratory of Biochemistry and Molecular Biology, School of Marine Sciences, Ningbo University, Ningbo 315832, China

**Keywords:** TCF19, transcription factor, H3K4me3, cancer, therapeutic target

## Abstract

Transcription factor 19 (TCF19) is a multifunctional biomolecule located within the major histocompatibility complex (MHC) class I region on chromosome 6p21.3. Structurally, TCF19 contains a plant homeodomain (PHD) finger that recognizes histone H3 lysine 4 trimethylation (H3K4me3) and a forkhead-associated (FHA) domain with yet-uncharacterized functions. Emerging evidence positions TCF19 as a multifunctional regulator associated with cell cycle progression, transcriptional regulation, cancer progression, and immune modulation through epigenetic and signaling mechanisms. This review provides the first systematic synthesis of TCF19’s structural domains, regulatory networks, and context-dependent functions across cancer and non-cancer diseases. We highlight critical knowledge gaps, including the unresolved function of its FHA domain and the lack of direct small-molecule inhibitors. In cancer, TCF19 drives proliferation, metastasis, immune evasion, and therapy resistance. Beyond cancer, TCF19 is involved in metabolic diseases, chronic infections, inflammatory disorders, and sensory deficits. TCF19 serves as a promising molecular biomarker for cancer diagnosis, prognosis, and treatment response monitoring, though direct targeting strategies remain unavailable.

## 1. Introduction

Epigenetics encompasses heritable changes in gene expression without alterations in the DNA sequence [1,2], mediated by key mechanisms including DNA methylation, histone modifications, chromatin remodeling and non-coding RNA-mediated regulation, which allow cells to dynamically adapt to environmental cues, orchestrate development, and maintain tissue-specific gene programs [3]. These processes are pivotal in directing immune responses, most notably in regulating the expression of major histocompatibility complex (MHC) molecules [4]. Within the MHC class I region on human chromosome 6p21.3 lies the gene encoding transcription factor 19 (TCF19) [5,6]. Unlike typical MHC-resident genes, TCF19 has been proposed to modulate chromatin accessibility and the transcriptional efficiency of MHC-I genes based on its chromatin-binding properties [7,8].

Since its initial localization within the MHC-I region in 1995 [7], research on TCF19 has expanded significantly across epigenetics, immunology, and oncology. However, the literature remains fragmented. Critical knowledge gaps persist: its pro-proliferative mechanisms in cancer [9] and its metabolic regulatory functions in diabetes are discussed in isolation without a unified cross-disease framework [8]. Although its direct pathogenic role in psoriasis has been excluded [10,11,12,13], and emerging pathways such as the TCF19-TRIM14-IFN-β axis have not been comprehensively reviewed. Moreover, direct small-molecule inhibitors of TCF19 are lacking, and no structure-guided drug development has been reported [14]. Critical synthesis is urgently needed to bridge these gaps and connect disparate research territories.

As a multifunctional protein containing both an epigenetic reader domain (plant homeodomain (PHD) finger) and a transcriptional regulatory module (forkhead-associated (FHA) domain) [8], TCF19 serves as an ideal model for deciphering “genetic–epigenetic–environmental” crosstalk. Its regulatory network offers novel insights into the molecular underpinnings of complex diseases [15]. TCF19 overexpression in hepatocellular carcinoma [16], lung adenocarcinoma [17], and other malignancies correlates with poor prognosis, suggesting its role as a pan-cancer biomarker, while its regulation of β-cell survival in diabetes highlights its therapeutic targeting value [18,19]. Integrating these findings is crucial for accelerating precision medicine strategies targeting TCF19-associated pathways.

Therefore, this review elucidates TCF19’s structure–function relationships, systematically summarizes its context-dependent roles in disease, and explores associated signaling pathways. These insights will clarify TCF19’s pathogenic mechanisms and evaluate its potential as a diagnostic biomarker and therapeutic target across interrelated diseases.

## 2. Structure and Function of TCF19

TCF19 was first identified in 1991 as a stringently growth-regulated gene, with mRNA expression peaking at the G1/S phase boundary [20]. Its deduced protein sequence contains a proline-rich domain, suggestive of a role as a transcriptional trans-activator. The human TCF19 gene is located within the HLA class I region on chromosome 6p21.3, approximately 130 kb telomeric to the HLA-C locus and positioned between HLA-C and corneodesmosin (CDSN) [7,12]. The gene spans three exons and two introns, with all exon-intron boundaries conforming to consensus splice signals. Notably, a 0.3 kb segment within intron 1 shows 82% homology to an Alu transposable element [7]. The transcriptional orientation of TCF19 is from telomere to centromere, which is opposite to that of its neighboring genes (HLA-C and CDSN). It encodes a protein of 345 amino acids.

**Figure 1:**
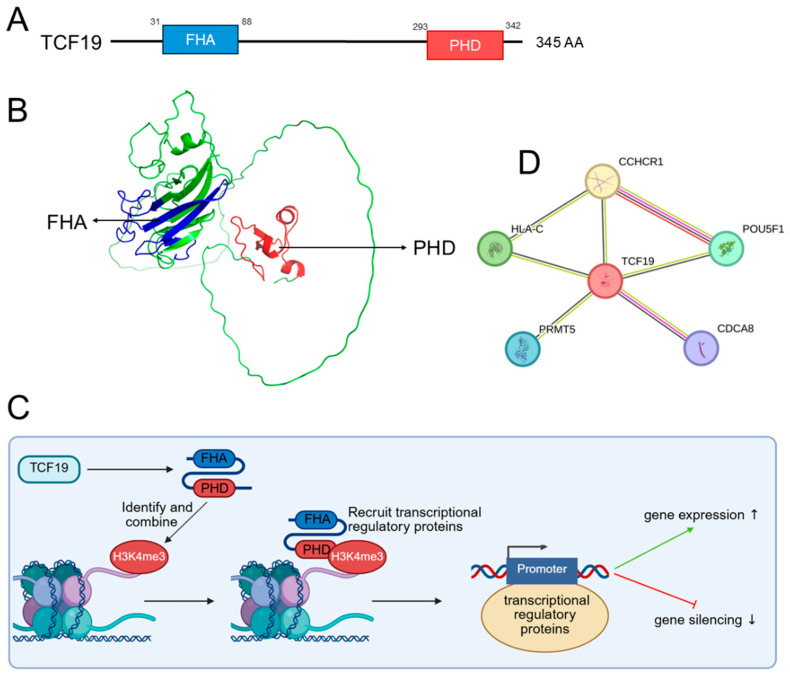
Structure and functional mechanism of TCF19. (**A**) Key genomic and protein features of TCF19. (**B**) Predicted three-dimensional structure of TCF19, modeled by AlphaFold, highlighting the forkhead-associated (FHA) domain (blue) and the plant homeodomain (PHD) finger (red). (**C**) Schematic of TCF19’s core functional mechanism. (**D**) STRING analysis of TCF19 protein–protein interaction network (confidence score ≥ 0.7) (https://string-db.org). The network shows predicted functional partners of TCF19, including POU5F1, CCHCR1, HLA-C, PRMT5, and CDCA8. Abbreviations: AA, amino acid; FHA, forkhead-associated domain; H3K4me3, histone H3 lysine 4 trimethylation; PHD, plant homeodomain finger; TCF19, transcription factor 19. CCHCR1, coiled-coil alpha-helical rod protein 1, HLA-C, HLA class I histocompatibility antigen, C, POU5F1, POU domain, class 5, transcription factor 1, PRMT5, protein arginine N-methyltransferase 5, CDCA8, cell division cycle associated 8.

TCF19 is defined by two key domains: a PHD finger and an FHA domain (Figure 1A,B). The PHD finger is a zinc-binding module that specifically recognizes histone H3 lysine 4 trimethylation (H3K4me3) via an aromatic cage formed by conserved tryptophan residues (W307 and W316). Structural integrity, dependent on zinc-coordinating residues (e.g., C324), is critical for binding; mutations at W316 or W307 abolish or weaken H3K4me3 affinity, respectively [8,21]. Functionally, this domain recruits epigenetic complexes (e.g., NuRD/HDAC1) to bidirectionally regulate downstream genes, thereby driving cell cycle progression, metabolic reprogramming, and cancer pathogenesis (Figure 1C). In contrast, the role of the TCF19 FHA domain remains largely uncharacterized. Canonical FHA domains are 80–100 amino acid phospho-threonine (pThr)-binding modules where specificity is dictated by residues adjacent to the pThr, particularly at the + 3 position [22,23]. They mediate phosphorylation-dependent signaling (e.g., DNA damage response) and phosphorylation-independent interactions, often by stabilizing oligomeric complexes and protecting phospho-sites from phosphatases, playing crucial roles in immune modulation and stress responses [24,25,26]. For TCF19, the FHA domain remains functionally uncharacterized, and its specific molecular ligands, binding partners, and broader biological functions await definitive elucidation [8]. We hypothesize that the FHA domain may recognize phospho-threonine marks on upstream kinases (e.g., AKT, ERK) in response to growth factor or stress signals or alternatively serve as a phosphorylation-independent scaffolding module to stabilize TCF19-containing complexes. Future structural determination and phospho-peptide library screening are needed to test these possibilities. To further illustrate the potential interaction landscape of TCF19, we performed a STRING analysis (Figure 1D). The network identifies several predicted functional partners of TCF19, including POU5F1, CCHCR1, HLA-C, PRMT5, and CDCA8.

## 3. TCF19 Regulatory and Signaling Networks

**Figure 2:**
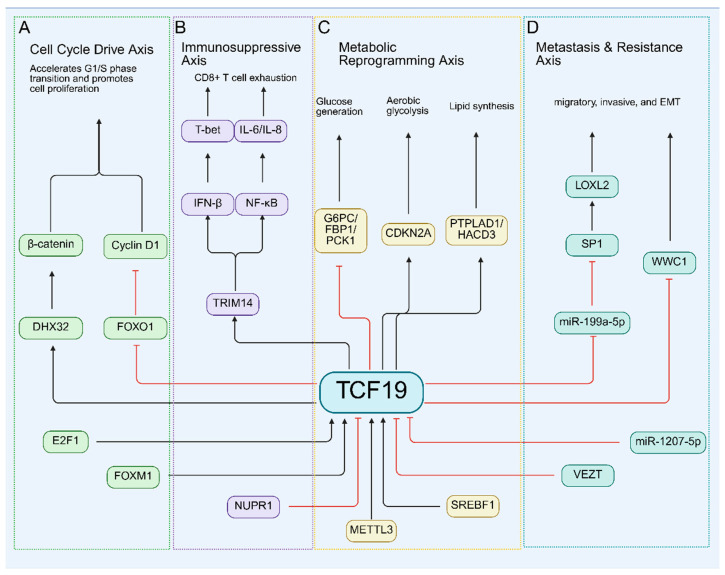
Regulatory network of TCF19. TCF19 is centrally positioned within a multi-layered regulatory network. Its expression is modulated by upstream transcription factors (activating: E2F1, FOXM1, SREBF1; repressing: NUPR1, VEZT) and post-transcriptional regulators (miR-1207-5p, METTL3/YTHDF1-mediated m^6^A modification). Downstream, TCF19 orchestrates four major oncogenic and metabolic axes: (**A**) Cycle Drive Axis: Accelerates the G1/S transition via FOXO1 suppression and Cyclin D1 upregulation. (**B**) Immunosuppressive Axis: Induces CD8^+^ T-cell exhaustion via the TRIM14-IFN-β signaling cascade. (**C**) Metabolic Reprogramming Axis: Suppresses gluconeogenic genes (G6PC/FBP1/PCK1), activates glycolysis (via CDKN2A), and regulates lipid synthesis (PTPLAD1/HACD3). (**D**) Metastasis and Resistance Axis: Promotes epithelial–mesenchymal transition (EMT) and invasion via the miR-199a-5p/SP1/LOXL2 cascade and WWC1 suppression. Abbreviations: CDKN2A, cyclin-dependent kinase inhibitor 2A; EMT, epithelial–mesenchymal transition; FOXM1, forkhead box M1; FOXO1, forkhead box O1; HACD3, 3-hydroxyacyl-CoA dehydratase 3; IFN-β, interferon-beta; LOXL2, lysyl oxidase homolog 2; METTL3, methyltransferase 3; NUPR1, nuclear protein 1; PTPLAD1, protein tyrosine phosphatase-like A domain containing 1; SREBF1, sterol regulatory element-binding transcription factor 1; TCF19, transcription factor 19; TRIM14, tripartite motif containing 14; VEZT, vezatin, adherens junctions transmembrane protein; WWC1, WW and C2 domain containing 1; YTHDF1, YTH N6-methyladenosine RNA-binding protein F1.

The functional activity of the transcription factor TCF19 is dynamically regulated through multi-layered mechanisms and, in turn, modulates downstream networks, establishing a complex signaling node. Its expression is bidirectionally modulated at the transcriptional level by activators like E2F1 and repressors such as VEZT and NUPR1. Concurrently, post-transcriptional regulation occurs via miRNA targeting (e.g., miR-1207-5p) and METTL3-catalyzed, YTHDF1-recognized m^6^A RNA methylation, and may be influenced by genetic variants (e.g., the SREBF1 binding site polymorphism). Functionally, TCF19 governs cell cycle progression, metabolic reprogramming, and immune microenvironment remodeling, particularly the induction of CD8^+^ T cell exhaustion. It also regulates metastasis-associated pathways such as epithelial–mesenchymal transition (EMT). This control is exerted through direct promoter binding of target genes (e.g., FOXO1, DHX32, TRIM14, CDKN2A, and gluconeogenic genes G6PC, FBP1, PCK1) or via regulation of non-coding RNAs (e.g., miR-199a-5p). Dysregulation of this intricate TCF19-centered network constitutes a core molecular mechanism driving malignant progression and metabolic disorders (Figure 2).

### 3.1. Upstream Regulatory Molecules

#### 3.1.1. E2F1

TCF19 shares a bidirectional promoter with its adjacent gene CCHCR1. This promoter is directly regulated by the transcription factor E2F1, a key G1/S-phase regulator. During the G1/S transition, E2F1 binds to this promoter region to synchronously activate transcription of both genes [15]. Functional studies demonstrate that a minimal 287 bp intergenic sequence is sufficient for this E2F1-mediated core co-induction, while full expression of CCHCR1 requires additional enhancer elements from both TCF19 and CCHCR1’s own exon 1 [15]. This mechanism illustrates how E2F1 orchestras the direct transcriptional co-activation of neighboring genes at critical cell cycle checkpoints, offering new insights into TCF19’s role and genomic regulation (Figure 2A).

#### 3.1.2. VEZT

VEZT functions as a transcriptional repressor that directly suppresses TCF19 expression. In the human gastric cancer cell line MKN-45, VEZT overexpression significantly downregulates TCF19 at the transcriptional level by inhibiting its promoter activity [27]. Functionally, TCF19 acts as a key downstream effector of VEZT, where overexpression drives G2/M phase transition and enhances cancer cell proliferation. Conversely, VEZT indirectly restrains gastric cancer cell growth and invasion by repressing TCF19 [27]. This established VEZT-TCF19 axis elucidates the core mechanism underlying VEZT’s tumor-suppressive role and offers novel insights for targeted therapies in gastric cancer (Figure 2D).

#### 3.1.3. SREBF1

In head and neck squamous cell carcinoma (HNSCC), a non-coding genetic variant modulates TCF19 expression through altered transcription factor binding. Specifically, the variant rs3131018 at chromosome 6p21.33 and its highly linked locus rs3094187 alter the binding affinity of the transcription factor SREBF1 to the TCF19 promoter. Biochemical assays confirm that allelic differences at rs3094187 modify SREBF1 binding efficiency, leading to significant changes in TCF19 expression levels [28]. This regulatory mechanism ultimately activates oncogenic signaling pathways, enhancing proliferation and migration in SCCHN cells (Figure 2C).

#### 3.1.4. NUPR1

The transcriptional repressor NUPR1 directly binds to the TCF19 promoter to suppress its activity and negatively regulate TCF19 expression. In pancreatic islets of Nupr1-knockout mice, both mRNA and protein levels of TCF19 are significantly elevated, leading to enhanced β-cell proliferation and upregulation of cell cycle genes like Ccna2. Conversely, NUPR1 overexpression inhibits TCF19 promoter activity [29]. This regulatory axis is physiologically critical: the upregulation of TCF19 is a key mechanism driving the expanded β-cell mass observed in Nupr1-deficient mice, which contributes to normoglycemia and protects against high-fat diet-induced metabolic defects (Figure 2B).

#### 3.1.5. FOXM1

Studies reveal that the transcription factor FoxM1, a key driver of cell proliferation, significantly upregulates TCF19 expression in pancreatic β-cells. FoxM1 overexpression induces a 12.8-fold increase in TCF19 mRNA in mouse islets and a 10.1-fold increase in human islets. Conversely, serum starvation reduces TCF19 expression by 89% in INS-1 cells [30]. This regulatory relationship demonstrates that FoxM1 promotes cell cycle progression—notably the G1/S transition—by activating TCF19, thereby critically influencing β-cell proliferation and survival (Figure 2A).

#### 3.1.6. miR-1207-5p

Studies demonstrate that miR-1207-5p negatively regulates TCF19 expression by directly binding to its 3′ untranslated region (3′UTR), thereby suppressing its transcriptional activity. In liver cancer, downregulation of miR-1207-5p leads to TCF19 overexpression. This, in turn, activates the Wnt/β-catenin signaling pathway, promoting β-catenin nuclear translocation and upregulating downstream oncogenes such as c-Myc and survivin (BIRC5), ultimately driving hepatocellular carcinoma (HCC) cell proliferation, migration, and invasion [9]. Clinical tissue analysis confirms a significant inverse correlation between high TCF19 and low miR-1207-5p expression in HCC, underscoring the pathological relevance of this regulatory axis [9] (Figure 2D).

#### 3.1.7. METTL3 and YTHDF1

METTL3, the core catalytic subunit of the N^6^-methyladenosine (m^6^A) methyltransferase complex, adds m^6^A marks to RRACH motifs within mRNA. In ulcerative colitis (UC), TCF19 expression is significantly upregulated and shows a strong positive correlation with METTL3 levels. Stimulating intestinal cells with the key inflammatory cytokine interferon-gamma (IFN-γ) markedly increases METTL3 expression and elevates global m^6^A methylation, indicating that inflammation promotes m^6^A modification, which influences downstream targets including TCF19 [31]. YTHDF1, an m^6^A reader protein that enhances the translation of modified mRNAs, is predicted by the TREW database to directly interact with TCF19 mRNA. However, this interaction awaits experimental validation [31]. This suggests YTHDF1 likely binds m^6^A sites on TCF19 mRNA to potentiate its translation [31]. Collectively, This METTL3/m^6^A/YTHDF1 axis represents a proposed post-transcriptional mechanism that may upregulate TCF19, based on in silico predictions and co-expression analyses (Figure 2C).

### 3.2. Downstream Effector Network

#### 3.2.1. FOXO1

TCF19 suppresses FOXO1 through direct and indirect mechanisms to regulate cancer cell cycle progression. In NSCLC, TCF19 directly binds the P1 region of the FOXO1 promoter to repress transcriptional activity [17]. In HCC, TCF19 activates the AKT pathway, inducing AKT-mediated phosphorylation of FOXO1 and subsequent cytoplasmic translocation and ubiquitin-dependent degradation [32]. Both pathways converge to downregulate FOXO1-targeted cell cycle inhibitor proteins p21, p27, p57 and upregulate cyclin D1, thereby accelerating G1/S phase transition and promoting cancer cell proliferation and tumor progression (Figure 2A).

#### 3.2.2. DHX32

In glioma, TCF19 directly binds to the DHX32 promoter to enhance its transcription, thereby activating the β-catenin signaling pathway. TCF19 overexpression significantly upregulates DHX32 expression, accelerating glioma cell proliferation and cell-cycle progression while suppressing apoptosis. Conversely, TCF19 knockdown inhibits DHX32 expression, impairs proliferation, induces cell cycle arrest, and promotes apoptosis. Functional rescue experiments confirm that DHX32 overexpression fully reverses the inhibitory effects caused by TCF19 knockdown on proliferation, cell cycle, and apoptosis [33]. This establishes the TCF19-DHX32-β-catenin axis as a critical oncogenic driver in glioma (Figure 2A).

#### 3.2.3. G6PC/FBP1/PCK1

TCF19 specifically recognizes histone H3 lysine 4 trimethylation (H3K4me3) via its PHD finger, an interaction enhanced under high-glucose conditions. It subsequently recruits the Nucleosome Remodeling and Deacetylase (NuRD) complex to the promoters of key gluconeogenic genes (G6PC, FBP1, PCK1). NuRD represses transcription by deacetylating histones, reducing marks such as H4K5Ac and H3K9Ac, leading to chromatin compaction [8]. This repression is amplified by insulin signaling promotes TCF19 enrichment at these promoters via insulin response elements (IREs), further suppressing gluconeogenic gene expression. Functionally, TCF19 knockdown in hepatocytes derepresses these genes and increases glucose production, whereas TCF19 overexpression suppresses endogenous glucose output [8]. Collectively, this establishes TCF19 as a critical epigenetic integrator of nutrient and hormonal signals to control hepatic glucose homeostasis (Figure 2C).

#### 3.2.4. TRIM14

In microsatellite-instable (MSI) endometrial cancer, TCF19 significantly upregulates TRIM14 transcription by directly binding to its promoter. The elevated TRIM14 protein then interacts with NEMO and TBK1, co-activating two parallel pathways: (1) it promotes TBK1 phosphorylation, leading to IRF3 activation and sustained overproduction of type I interferon IFN-β; (2) it activates the NF-κB pathway by inducing IκBα phosphorylation and degradation, stimulating pro-inflammatory cytokines (e.g., IL-6, IL-8) [14]. This persistent, TCF19-TRIM14-driven IFN-β signaling reprograms CD8^+^ T cell differentiation. It upregulates exhaustion-related transcription factors (TOX, T-bet) while suppressing the stemness-maintaining TCF7-BCL6 axis, culminating in the functional exhaustion of tumor-infiltrating CD8^+^ T cells [14]. Critically, within the mismatch repair-deficient (MMRd) MSI tumor microenvironment, this axis synergizes with endogenous cGAS-STING pathway activation to further amplify IFN-β production, creating a feed-forward loop that exacerbates immunosuppression and tumor progression [34] (Figure 2B).

#### 3.2.5. CDKN2A

In osteosarcoma, TCF19 directly binds to and enriches the CDKN2A promoter, enhancing its expression. This TCF19-driven CDKN2A upregulation exerts dual pro-tumorigenic effects. First, it activates the glycolytic pathway within tumor cells, evidenced by an increased extracellular acidification rate and elevated glycolysis-related protein expression. Second, it induces M2 polarization of co-cultured macrophages, characterized by an increased CD206^+^/CD86^−^ ratio, elevated IL-10 secretion, and reduced IL-12 secretion [35]. Together, these TCF19/CDKN2A axis-mediated events—metabolic reprogramming and immune microenvironment remodeling—promote osteosarcoma malignant progression. This includes enhanced proliferation, migration, invasion, and in vivo tumor growth (Figure 2C).

#### 3.2.6. miR-199a-5p/SP1/LOXL2

TCF19 is identified as a critical epithelial–mesenchymal transition (EMT)-associated gene in breast cancer with diagnostic and prognostic significance. Mechanistically, TCF19 downregulates miR-199a-5p, thereby relieving its inhibitory effect on the transcription factor SP1. This leads to nuclear enrichment of SP1 and enhanced binding to the LOXL2 promoter, culminating in significant LOXL2 upregulation [36]. The TCF19/miR-199a-5p/SP1/LOXL2 axis facilitates the migratory, invasive, and EMT activities of breast cancer cells in vitro. In vivo subcutaneous and tail-vein injection models further confirm its oncogenic and metastatic potential by promoting primary tumor growth and metastatic establishment [36]. This delineates a coherent signaling cascade through which TCF19 drives breast cancer progression and metastasis (Figure 2D).

#### 3.2.7. WWC1

Studies indicate that TCF19 exacerbates colorectal cancer (CRC) progression through the negative regulation of the tumor suppressor WWC1. TCF19 is significantly upregulated while WWC1 is downregulated in CRC tissues and cells. Clinically, high TCF19 or low WWC1 expression predicts poor patient survival, and TCF19 levels positively correlate with distant metastasis [37]. Functionally, silencing TCF19 in HT29 cells inhibits proliferation, colony formation, and migration, while its overexpression in HCT-8 cells exerts pro-tumorigenic effects. Molecularly, TCF19 directly suppresses WWC1 expression in CRC. Critically, rescue experiments show that WWC1 knockdown abolishes the regulatory effects of TCF19 on CRC cell malignancy [37]. Consequently, TCF19 drives CRC progression and poor prognosis by suppressing WWC1 to promote proliferation, migration, and metastatic potential (Figure 2D).

#### 3.2.8. PTPLAD1/HACD3

TCF19 dynamically regulates the expression of the fatty acid elongase gene PTPLAD1 (encoding HACD3) in response to palmitic acid (PA)-induced metabolic stress. Under basal conditions, TCF19—functioning as an H3K4me3-binding protein—forms a complex with the transcription factor TCF7L2 that is co-recruited to the PTPLAD1 promoter, repressing its expression. Under PA stress, however, reduced H3K4me3 enrichment at the promoter leads to the dissociation of the TCF19-TCF7L2 complex, resulting in PTPLAD1 gene activation and increased HACD3 expression [38]. This regulatory shift is clinically relevant: gene expression analyses from PA-injected mice and patients with non-alcoholic fatty liver disease (NAFLD) show an inverse correlation where decreased TCF7L2 expression correlates with increased HACD3 expression. The consequent upregulation of HACD3 enhances fatty acid chain elongation and triglyceride production, thereby promoting the progression of metabolic dysfunction-associated steatotic liver disease (MASLD) [38]. Thus, TCF19, in concert with TCF7L2 and responsive to H3K4me3 dynamics, plays a pivotal role in lipid metabolism and metabolic disorders (Figure 2C).

## 4. The Role of TCF19 in Homeostasis

As a multifunctional regulator, TCF19 integrates diverse biological processes through epigenetic and transcriptional mechanisms. It serves as a regulator of cellular proliferation and the cell cycle, orchestrates transcriptional programs, influences developmental pathways, and serves as a central hub for metabolic homeostasis and stress adaptation.

### 4.1. TCF19 Regulates Cell Cycle and Proliferation

TCF19 functions as a critical cell growth regulatory factor, with its expression peaking at the G1/S phase transition of the cell cycle to directly modulate proliferation [13]. In the INS-1 insulinoma cell model, *TCF19* knockdown downregulates key cell cycle genes (e.g., Cyclin E, A, and B), induces G1/S phase arrest, and significantly reduces proliferation [30]. Conversely, TCF19 overexpression in MKN-45 gastric cancer cells accelerates progression through the G2/M phase [27]. Beyond cycle control, TCF19 promotes cell survival by suppressing both baseline and endoplasmic reticulum (ER) stress-induced apoptosis, thereby maintaining a cellular balance that favors proliferation [30].

### 4.2. TCF19 Regulates Transcription

TCF19 mediates transcriptional regulation primarily through its PHD finger, which specifically binds H3K4me3 to recruit epigenetic complexes [8,21]. It exhibits dual, context-dependent regulatory functions: it binds the P1 region of the FOXO1 promoter to suppress its activity [32], while it binds the DHX32 promoter to enhance transcription, thereby activating the β-catenin signaling pathway [33]. Genomically, TCF19 shares a bidirectional promoter with its neighboring gene CCHCR1. This promoter is activated by the G1/S-phase transcription factor E2F1, leading to co-induction of both genes during the G1/S transition [15]. Furthermore, a TCF19 splice variant (TCF19-D) shares two exons and one intron with the adjacent POU5F1 variant OCT4B3 at identical genomic coordinates. This antisense overlap produces complementary transcripts that may mutually regulate stability or splicing through RNA-RNA pairing. The shared genomic regions show low evolutionary conservation, suggesting human-specific rapid evolution [39].

## 5. Role of TCF19 in Diseases

**Figure 3:**
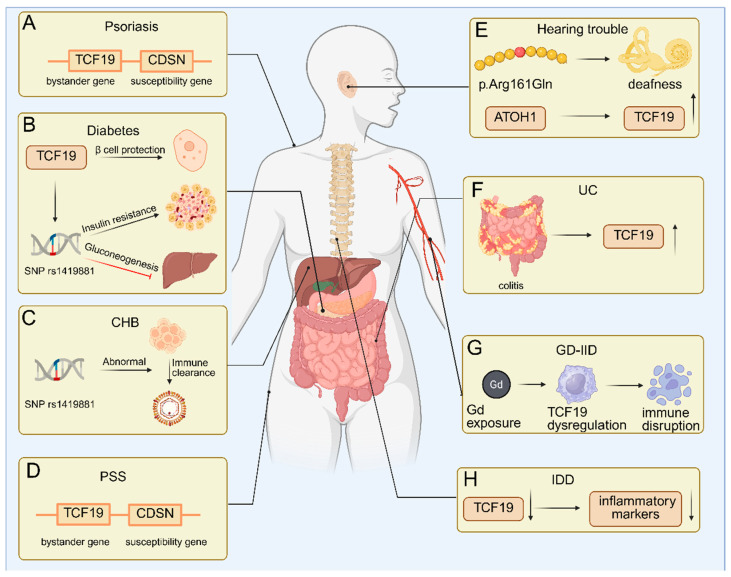
Role of TCF19 in diseases. TCF19 exhibits a spectrum of involvement in non-cancerous pathologies. (**A**) In psoriasis, it acts as a bystander gene within the PSORS1 locus, lacking pathogenic variants or phenotypic consequences upon deletion, where CDSN is the primary susceptibility gene. (**B**) In diabetes, TCF19 protects β-cells from apoptosis and endoplasmic reticulum stress, and its genetic variants are linked to insulin resistance. (**C**) In chronic hepatitis B (CHB), polymorphisms such as rs1419881 modulate immune-mediated viral clearance. (**D**) In peeling skin disease (PSD), TCF19 deletion does not contribute to pathology, which is driven by loss-of-function mutations in CDSN. (**E**) A missense variant (p.Arg161Gln) in TCF19 causes hearing loss, potentially by disrupting cochlear development regulated by ATOH1. TCF19 is also implicated in (**F**) ulcerative colitis (UC), (**G**) gadolinium deposition-induced immunometabolism dysregulation (GD-IID), and (**H**) intervertebral disk degeneration (IDD), where it promotes inflammation and tissue degeneration. Abbreviations: ATOH1, atonal BHLH transcription factor 1; CDSN, corneodesmosin; CHB, chronic hepatitis B; GD-IID, gadolinium deposition-induced immunometabolism dysregulation; IDD, intervertebral disk degeneration; PSD, peeling skin disease; SNP, single-nucleotide polymorphism; TCF19, transcription factor 19; UC, ulcerative colitis.

Beyond its core physiological functions, TCF19 exhibits pleiotropic contributions to human disease through genetic susceptibility, epigenetic dysregulation, and tissue-specific functional perturbations. Its pathogenic involvement spans a broad clinical spectrum, including metabolic diseases (diabetes), chronic infections (chronic hepatitis B, CHB), rare genetic syndromes (peeling skin disease, PSD), sensory impairment (hearing loss), and inflammatory conditions (ulcerative colitis, UC), underscoring its multifaceted impact on human pathology (Figure 3).

### 5.1. Psoriasis

Psoriasis vulgaris is a chronic, immune-mediated inflammatory skin disease characterized by dysregulated keratinocyte proliferation and defective differentiation, resulting in thickened, scaly plaques primarily driven by genetic susceptibility and environmental triggers [40]. TCF19 was initially considered a candidate gene due to its genomic position within the major psoriasis susceptibility locus PSORS1 (6p21.3) and its role in cell cycle regulation [11]. Subsequent fine-mapping delimited the critical region to a 111 kb segment telomeric to HLA-C (89–200 kb) encompassing TCF19, POU5F1, and CDSN. Despite this location, TCF19’s functional profile shows no direct link to core psoriatic pathology, such as keratinocyte hyperproliferation and inflammation. In contrast, the skin-specific gene CDSN emerged as a stronger etiological candidate due to its cutaneous expression and biological relevance [13]. Definitive genetic analyses found no psoriasis-associated polymorphisms in TCF19’s coding or regulatory regions [12], and comparative haplotype sequencing revealed no risk-specific variants at the mRNA or protein level [41], formally excluding it as the causal PSORS1 gene. Clinical evidence further supports this: a patient with a genomic deletion encompassing TCF19 exhibited peeling skin syndrome without psoriatic phenotypes, confirming its non-essential role in psoriasis pathogenesis [10]. Consequently, TCF19 is now established as a pathogenically inert “bystander gene” within the PSORS1 locus, with CDSN and CCHCR1 identified as the principal drivers of disease through keratinocyte-specific mechanisms (Figure 3A) [42,43].

### 5.2. Diabetes

Type 1 diabetes (T1D) is a chronic autoimmune disease characterized by immune-mediated destruction of pancreatic β-cells [44]. TCF19 contributes to T1D susceptibility through a four-locus epistatic interaction with *MUC21*, *MUC22*, and *PSORS1C1* [45]. It resides within the conserved ancestral haplotype AH18.2, which is associated with T1D risk, suggesting haplotype-driven effects on predisposition [45]. Immunologically, TCF19 is highly expressed in B-cell progenitors and germinal center cells, where it may sustain immune homeostasis by modulating the balance between autoreactive and regulatory T cells, potentially mitigating autoimmune attack on β-cells [46]. At the β-cell level, TCF19 is islet-enriched and correlates with obesity. Its knockdown impairs cell cycle and promotes ER stress-induced apoptosis [19,30], while its overexpression activates DNA repair and antiviral pathways, suggesting a protective role under stress (Figure 3B) [18].

Type 2 diabetes (T2D) is a metabolic disorder driven by insulin resistance and progressive β-cell dysfunction [47]. The SNP rs3130501 near the TCF19 locus confers T2D risk by enhancing insulin resistance and postprandial hyperglycemia [48,49]. In the liver, TCF19 represses gluconeogenic genes (G6PC, FBP1, PCK1) to suppress hepatic glucose output [8]. In β-cells, TCF19 promotes proliferation and survival [29]. Its knockdown induces G1/S arrest and exacerbates ER stress-induced apoptosis, while overexpression activates DNA damage repair pathways [19,30]. Thus, TCF19 sustains β-cell mass to compensate for insulin resistance in T2D but does not modulate insulin secretion, enabling metabolic compensation until decompensation occurs (Figure 3B) [8,30]. It is worth noting that the same TCF19-driven proliferative signal that promotes oncogenesis in cancer cells is harnessed for β-cell mass expansion in diabetes, illustrating a context-dependent functional duality that remains mechanistically unresolve.

### 5.3. Chronic Infections

Chronic hepatitis B (CHB) is a persistent HBV infection that can progress to liver disease [50]. Genome-wide association studies (GWAS) have identified several TCF19 variants associated with CHB susceptibility. SNP rs1419881 in the 3′UTR of TCF19 significantly increases CHB risk, potentially affecting mRNA stability or translation [51]. Another study reported a suggestive association for rs1419881 [52]. However, a study in a Thai population found no association, suggesting possible population-specific effects [53]. Moreover, a SNP rs7453920, located near HLA loci on 6p21.32, is a key host factor influencing HBV persistence independently of classic HLA alleles [54]. A 2017 study noted that the nearby OCT4 variant rs1265163 might exert stronger independent effects, though TCF19 remains a critical marker within this susceptibility region [55]. Collectively, TCF19 is a plausible genetic modulator of CHB susceptibility, but further multi-population studies are needed for definitive validation (Figure 3C).

### 5.4. Rare Genetic Syndromes

Peeling skin syndrome (PSS) is a rare autosomal recessive ichthyosis characterized by continuous exfoliation. It comprises acral and generalized forms, with the latter subdivided into non-inflammatory (type A) and inflammatory (type B) variants; type B is now designated PSD [56]. PSD, an autosomal recessive disorder caused by CDSN mutations, is characterized by superficial peeling of the upper epidermis. CDSN is a major component of corneodesmosomes that plays an important role in maintaining epidermis integrity [57]. Notably, large genomic deletions in PSD patients on chromosome 6p21.3 frequently encompass the TCF19 locus (e.g., 59.1 kb deletions covering both CDSN and TCF19 [58] or 49-72 kb deletions involving TCF19’s 5′ end [56]). However, the clinical phenotype is solely attributable to CDSN loss-of-function. Patients present with classic PSD symptoms—skin exfoliation and inflammation—but none exhibit hallmarks of TCF19-linked disorders like T1D [56,58]. This confirms that TCF19 deletion does not produce additional symptoms and is not involved in PSD pathogenesis, while CDSN mutations are the established driver [58]. Consequently, TCF19 is considered a bystander gene in this context, though its potential biological roles in PSD warrant future functional investigation (Figure 3D).

### 5.5. Hearing Trouble

A heterozygous missense variant in TCF19 (c.482G>A; p.Arg161Gln) has been implicated in severe, non-syndromic hearing loss within an Iranian family (a mother and two sons). The affected arginine (Arg161) is evolutionarily conserved, and 3D modeling indicates the mutant glutamine (Gln161) disrupts hydrogen bonding, potentially altering protein conformation [59]. Functionally, TCF19 is highly expressed in murine cochlear connective tissue and regulated by vezatin, a protein critical for hair cell mechanotransduction [59]. It is also upregulated by Atonal BHLH transcription factor 1 (ATOH1), a key driver of hair cell regeneration, suggesting a role in auditory system development or maintenance. The p.Arg161Gln variant may thus impair TCF19-mediated transcriptional programs, disrupting cochlear structure or hair cell homeostasis to cause hearing loss [59]. Separately, TCF19 has been identified as a candidate for age-related hearing impairment (ARHI). Methylation levels at CpG sites near TCF19 significantly correlate with its blood expression, suggesting that epigenetic regulation of TCF19 expression may influence ARHI susceptibility (Figure 3E) [60].

### 5.6. Other Non-Cancer Diseases

Ulcerative colitis (UC) is a chronic inflammatory bowel disease characterized by persistent mucosal inflammation and ulceration, presenting with symptoms like diarrhea, bloody stools, and abdominal pain. Its etiology involves genetic susceptibility, immune dysregulation, and environmental factors [61]. TCF19 is significantly upregulated in UC, and its intronic SNP rs139102013 may contribute to pathogenesis via epigenetic regulation. This upregulation correlates with m^6^A RNA methylation machinery: TCF19 binds the m^6^A reader YTHDF1, and clinical samples show co-expression with the m^6^A writer METTL3, implicating m^6^A-dependent transcript stabilization in inflammation (Figure 3F) [31]. Collectively, this METTL3/m^6^A/YTHDF1 axis represents a postulated post-transcriptional mechanism potentially upregulating TCF19 in UC inflammation, although direct experimental validation of m^6^A modification on TCF19 mRNA has not yet been reported [31]. Beyond classic inflammatory disease, TCF19 dysregulation is implicated in iatrogenic and degenerative conditions. Prolonged retention of gadolinium-based contrast agents (GBCAs) like Dotarem in macrophages disrupts TCF19 expression, coinciding with SMAD3 abnormalities and impaired anti-inflammatory responses, positioning TCF19 as a central mediator in gadolinium deposition-induced immunometabolism dysregulation (GD-IID) (Figure 3G) [62,63]. Furthermore, TCF19 is a key upregulated gene in transitional-state nucleus pulposus cells during intervertebral disk degeneration (IDD). Integrated single-cell and bulk RNA-seq analyses identify its critical association with IDD progression, and in vivo experiments in rats demonstrate that TCF19 knockdown significantly alleviates degeneration by reducing abnormal collagen deposition and inflammatory markers (Figure 3H) [64].

## 6. Role of TCF19 in Cancer

**Figure 4:**
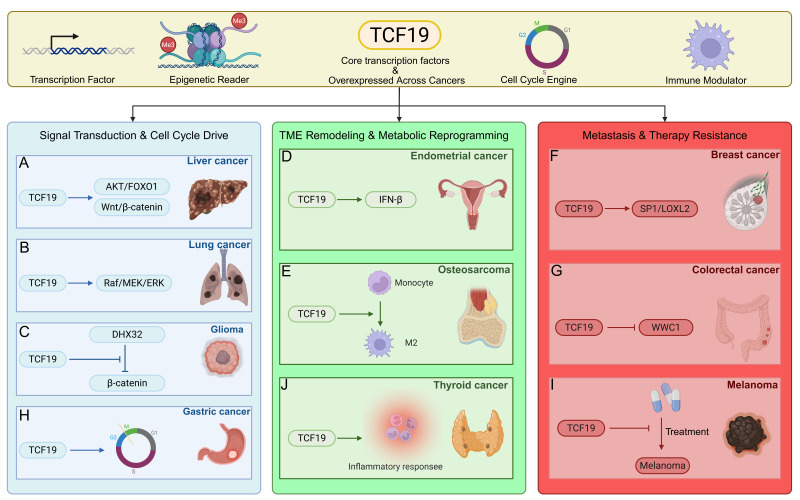
Oncogenic roles of TCF19. TCF19 is overexpressed in diverse malignancies and functions as a core oncogenic transcription factor. It drives tumor progression through three major mechanistic axes: (**A**–**C**,**H**) Signal Transduction and Cell Cycle Drive: In liver cancer, it activates the AKT/FOXO1 and Wnt/β-catenin pathways; in lung cancer, it activates Raf/MEK/ERK; in glioma, it upregulates DHX32 to activate β-catenin signaling. (**D**,**E**,**J**) Tumor Microenvironment Remodeling and Metabolic Reprogramming: In endometrial cancer, TCF19 induces interferon-beta (IFN-β) production, leading to CD8^+^ T-cell exhaustion; in osteosarcoma, it promotes M2 macrophage polarization; in thyroid cancer, it modulates inflammatory responses. (**F**,**G**,**I**) Metastasis and Therapy Resistance: In breast cancer, TCF19 promotes epithelial–mesenchymal transition (EMT) via the SP1/LOXL2 axis; in colorectal cancer, it enhances malignancy by suppressing WWC1; in melanoma, it confers resistance to targeted and immunotherapies. Abbreviations: AKT, AKT serine/threonine kinase; DHX32, DExH-box helicase 32; EMT, epithelial–mesenchymal transition; ERK, extracellular signal-regulated kinase; FOXO1, forkhead box O1; IFN-β, interferon-beta; LOXL2, lysyl oxidase homolog 2; MEK, MAPK/ERK kinase; TCF19, transcription factor 19; WWC1, WW and C2 domain containing 1.

TCF19 exhibits significantly elevated expression across diverse malignancies, including hepatocellular carcinoma, lung adenocarcinoma, glioma, endometrial, breast, colorectal, and gastric cancers. TCF19 has been implicated in tumor cell proliferation, survival, invasion, and metastasis, with mechanistic evidence from knockdown/rescue experiments in cell lines and xenograft models. These include core signaling pathways (AKT/FOXO1, Wnt/β-catenin, Raf/MEK/ERK, PLK1), epigenetic regulation via H3K4me3 recognition, cell cycle progression, metabolic reprogramming, tumor immune microenvironment remodeling, and epithelial–mesenchymal transition (EMT). Its aberrant expression correlates with poor patient prognosis and has emerged as a predictive biomarker for treatment response and a promising target for combination therapies (Figure 4 and Table 1). Among these pathways, AKT/FOXO1 and Wnt/β-catenin are the most consistently implicated across multiple cancer types and are considered the most relevant for diagnostic and therapeutic targeting. The Raf/MEK/ERK pathway is particularly prominent in lung cancer and melanoma, while PLK1 has been specifically linked to breast cancer.

### 6.1. Hepatocellular Carcinoma

TCF19 functions as a oncogene in hepatocellular carcinoma (HCC), the predominant primary liver cancer associated with CHB and cirrhosis [65,66]. It is significantly upregulated in HCC tissues and promotes tumor proliferation, in part, by activating the AKT/FOXO1 pathway: TCF19 overexpression downregulates cell cycle inhibitors (p57, p21, p27), upregulates cyclin D1, and enhances phosphorylation of Rb, FOXO1, and AKT, thereby driving the G1/S transition [32]. Its PHD finger specifically binds H3K4me3 to epigenetically regulate proliferation-related genes like CCND1 and FGF2; mutation of the critical residue W316 abrogates this function and suppresses tumor growth [21]. TCF19’s own expression is regulated post-transcriptionally by the lncRNA miR194-2HG/miR-1207-5p axis, with miR-1207-5p inhibiting TCF19 by binding its 3′UTR [9]. Functionally, TCF19 activates the Wnt/β-catenin pathway to facilitate β-catenin nuclear translocation and upregulate downstream oncogenes (e.g., c-Myc), enhancing HCC cell migration, invasion, and proliferation [9]. Furthermore, TCF19 interacts with the tumor suppressor p53 to co-regulate metabolic genes (e.g., TIGAR, SCO2), reprogramming mitochondrial energy metabolism and stress adaptation in HCC cells [67]. Clinically, high TCF19 expression correlates with poor prognosis in HCC patients [68] and confers resistance to the MEK inhibitor trametinib [69]. RNA-Seq data confirm that TCF19/p53-mediated metabolic regulation is essential for HCC cell survival [67], solidifying TCF19’s role as a critical driver and therapeutic target (Figure 4A). Thus, TCF19 serves as an independent prognostic biomarker for HCC, and its expression level may inform patient risk stratification.

### 6.2. Lung Cancer

Lung cancer, a malignant tumor arising from bronchial or alveolar epithelium, is the leading cause of global cancer incidence and mortality, primarily classified as non-small-cell lung cancer (NSCLC) and small-cell lung cancer [70,71]. TCF19 is markedly overexpressed in NSCLC subtypes, particularly lung adenocarcinoma and squamous cell carcinoma [17]. Mechanistically, TCF19 enhances cancer cell proliferation by accelerating the G1/S transition: it suppresses FOXO1 promoter activity, downregulating cell cycle inhibitors (p21, p27, p57) and upregulating cyclin D1 [17]. Furthermore, in vivo and in vitro studies demonstrate that TCF19 overexpression accelerates tumor progression by activating the Raf/MEK/ERK pathway. Specifically, TCF19 increases phosphorylation of Raf1, MEK1/2, and ERK1/2, while pharmacological inhibition of Raf1 or ERK reduces cell cycle protein expression and suppresses cancer cell growth (Figure 4B) [72]. TCF19 expression level is a potential prognostic indicator in NSCLC, with higher expression associated with shorter survival.

### 6.3. Glioma

Gliomas, the most common primary tumors of the central nervous system, arise from glial cells. They exhibit a wide spectrum of malignancy but are predominantly highly invasive and pose significant therapeutic challenges [73]. TCF19 functions as a key oncogenic factor in this context, with significant upregulation in glioma tissues correlating with poor clinical prognosis. It drives glioma cell proliferation and cell-cycle progression while suppressing apoptosis. Mechanistically, TCF19 binds to the DHX32 promoter to enhance its transcription, thereby activating the β-catenin signaling pathway to promote tumorigenesis (Figure 4C) [33]. TCF19 may serve as a prognostic biomarker for glioma, as its upregulation correlates with poor clinical outcomes.

### 6.4. Endometrial Cancer

Endometrial cancer, the most common malignancy of the uterine body, arises from the endometrium. It predominantly affects postmenopausal women, with unexpected vaginal bleeding serving as its hallmark warning sign [74,75]. TCF19 is significantly upregulated in MSI endometrial cancer and correlates with poor prognosis. Mechanistically, TCF19 facilitates TRIM14 transcription, which in turn activates the TBK1-IRF3 and NF-κB pathways. This cascade induces overproduction of interferon-beta (IFN-β), promoting CD8^+^ T cell exhaustion within the tumor microenvironment. Consequently, combined targeting of TCF19 and anti-PD-1 therapy synergistically enhances tumor control in humanized models by reversing T cell exhaustion (Figure 4D) [14]. In MSI endometrial cancer, TCF19 expression correlates with poor prognosis, positioning it as a candidate prognostic biomarker.

### 6.5. Osteosarcoma

Osteosarcoma is the most common primary malignant bone tumor, typically arising in the metaphysis of long bones in adolescents and young adults, characterized by cancer cells that produce immature bone [76,77]. TCF19 promotes osteosarcoma progression by establishing the TCF19-CDKN2A axis: it directly binds to and enriches at the CDKN2A promoter, enhancing its expression. This axis drives tumorigenesis by activating glycolysis and inducing M2 macrophage polarization within the tumor microenvironment. In vivo studies confirm that TCF19 knockdown suppresses CDKN2A expression, inhibits tumor growth and metabolic remodeling, thereby blocking malignant progression (Figure 4E) [35]. Although CDKN2A is classically recognized as a tumor suppressor, the TCF19-CDKN2A axis described here reveals a pro-tumorigenic role in osteosarcoma, likely through non-canonical effects on glycolysis and macrophage polarization, highlighting a surprising context-dependent function that warrants further investigation. These findings suggest that TCF19 has both prognostic and therapeutic value in osteosarcoma.

### 6.6. Breast Cancer

Breast cancer develops from cells in the breast tissue, most commonly in the milk ducts or lobules. It primarily affects women but can also occur in men; early detection through screening significantly improves outcomes, and many cases are driven by estrogen or HER2 [78,79]. TCF19 is significantly upregulated in breast cancer tissues [63] and promotes metastasis by negatively regulating miR-199a-5p [68]. This relief of suppression enhances the transcription factor SP1’s binding to the LOXL2 promoter, upregulating LOXL2 expression. This TCF19/miR-199a-5p/SP1/LOXL2 axis consequently drives epithelial–mesenchymal transition (EMT), migration, and invasion [36]. Additionally, TCF19 silencing inhibits the proliferation, invasion, migration, and tumorigenesis of breast cancer cells (e.g., MCF-7) by suppressing the PLK1 signaling pathway, a key driver of tumor progression [80]. TCF19 is also significantly upregulated following estrogen-progestogen therapy (EPT) compared to estrogen-only therapy (ET) and shows higher expression in tumors versus normal adjacent tissue, implicating it alongside CCNE2 and CDCA5 in potentially driving EPT-associated tumorigenesis [81]. However, elevated TCF19 expression shows no significant correlation with overall survival in ER-positive patients, suggesting its prognostic value may be context-dependent [81]. Collectively, TCF19 drives breast cancer metastasis via the miR-199a-5p/SP1/LOXL2 axis and fuels tumor growth through PLK1 activation, though its role as a prognostic biomarker requires further study (Figure 4F). Although the prognostic significance of TCF19 in breast cancer may be context-dependent, its elevated expression in metastatic disease suggests potential utility as a biomarker for aggressive tumors.

### 6.7. Colorectal Cancer

Colorectal cancer (CRC) develops from the inner lining of the colon or rectum, often originating as benign polyps that can undergo malignant transformation over years. It is strongly linked to age, lifestyle factors, and family history but is highly preventable through screening and treatable when detected early [82,83]. TCF19 is significantly upregulated in CRC tissues and cells, and its expression inversely correlates with levels of the tumor suppressor WWC1. Clinically, high TCF19 or low WWC1 expression predicts reduced survival and increased distant metastasis in CRC patients [37]. Functionally, TCF19 silencing suppresses tumor proliferation and migration, while its overexpression exacerbates malignant phenotypes. Mechanistically, TCF19 is associated with CRC progression and metastasis and may act through negative regulation of WWC1, based on expression correlation and rescue experiments (Figure 4G) [37]. High TCF19 or low WWC1 expression predicts reduced survival and increased distant metastasis, supporting TCF19 as a prognostic biomarker in CRC.

### 6.8. Gastric Cancer

Gastric cancer, most commonly adenocarcinoma, originates in the stomach lining and is strongly linked to helicobacter pylori infection, smoking, and dietary factors. It is often diagnosed at an advanced stage due to vague early symptoms, contributing to a relatively poor prognosis [84,85]. In this malignancy, TCF19 functions as an oncogene by promoting G2/M phase transition and enhancing cell cycle progression. Its expression is directly suppressed by the tumor suppressor VEZT, which transcriptionally represses TCF19. The established VEZT-TCF19 axis is a key regulator of gastric carcinogenesis, where VEZT suppresses tumor growth and invasion by downregulating TCF19 and its associated cell cycle genes (Figure 4H) [27]. TCF19 is a downstream effector of the tumor suppressor VEZT, and its expression correlates with gastric cancer progression, suggesting its potential as a prognostic indicator.

### 6.9. Melanoma

Melanoma is a highly aggressive skin cancer that develops from melanocytes, often triggered by intense UV exposure. While it can rapidly metastasize if not detected early, it has a near 100% cure rate when treated at its earliest stage, typically identified by changes in a mole’s size, shape, or color [86,87]. TCF19 is a key transcription factor in NRAS-mutant melanoma, with its high expression significantly correlating with poor patient survival and resistance to both targeted therapies and immunotherapy [88]. A promising therapeutic strategy involves the synergistic combination of BET and MEK inhibitors, which downregulates TCF19 expression. This disruption of TCF19-mediated regulation of cell cycle and apoptosis genes induces tumor cell apoptosis and overcomes therapeutic resistance. Clinically, TCF19 mRNA levels are significantly lower in treatment responders compared to non-responders, positioning TCF19 as a potential predictive biomarker for treatment response and a critical target for rational combination therapies (Figure 4I) [88].

### 6.10. Thyroid Cancer

Thyroid cancer involves abnormal cellular growth within the thyroid gland, ranging from benign nodules to malignant tumors. While most nodules are benign, malignant types are often slow-growing and highly curable, typically diagnosed with ultrasound and fine-needle aspiration [89,90]. TCF19 is significantly associated with malignant phenotypes in thyroid cancer and drives disease progression by regulating inflammatory and immune responses [91]. Intriguingly, the deleterious missense variant rs2073724 exhibits strong genetic associations with thyroid dysfunction, autoimmune thyroiditis, and thyroid cancer risk. Functionally, this variant disrupts TCF19’s ability to bind target gene promoters, thereby reversing its oncogenic effects [91]. Collectively, TCF19 represents a promising therapeutic target for aggressive thyroid cancer, with the rs2073724 variant serving as a clinically significant biomarker for disease susceptibility and functional impact (Figure 4J). The deleterious missense variant rs2073724 serves as a clinically significant biomarker for thyroid cancer susceptibility and may predict disease aggressiveness.

### 6.11. Prostate Cancer

Prostate cancer is a prevalent malignancy that progresses to metastatic disease in a subset of patients, accounting for significant mortality worldwide. A gene expression meta-analysis across multiple patient cohorts identified TCF19 as an epithelial gene significantly upregulated in metastatic prostate cancer, with prognostic potential and a negative association with androgen receptor activity [28]. Functional studies revealed that TCF19 is required for full metastatic capacity; its depletion impairs tumor growth and reduces vascular permeability, supporting a role in prostate tumor cell dissemination [28]. Thus, TCF19 represents a potential prognostic biomarker and therapeutic target for aggressive prostate cancer.

### 6.12. Head and Neck Squamous Cell Carcinoma (HNSCC)

HNSCC is one of the most common cancers worldwide, arising from the oral cavity, pharynx, and larynx. A genome-wide association study in a Chinese population identified three novel genetic variants associated with HNSCC risk, including rs3131018 at 6p21.33 [92]. Further analysis revealed that rs3094187, in high linkage disequilibrium with rs3131018, modifies TCF19 expression by altering the binding affinity of the transcription factor SREBF1 to the TCF19 promoter [92]. Functional assays demonstrated that TCF19 inhibition attenuates cell proliferation and migration in SCCHN cells and may affect multiple tumorigenesis-related pathways [92]. These findings position TCF19 as a putative susceptibility gene for HNSCC.

**Table 1:** Oncogenic functions of TCF19 across cancer types. This table outlines the tumor-promoting functions of TCF19, its key molecular targets/signaling pathways, and supporting references in various malignancies. Abbreviations: HCC (Hepatocellular carcinoma), NSCLC (Non-small-cell lung cancer), CRC (Colorectal cancer), EMT (Epithelial–mesenchymal transition), PLK1 (Polo-like kinase 1), WWC1 (WW and C2 domain containing 1), CDKN2A (Cyclin-dependent kinase inhibitor 2A), HNSCC (Head and Neck Squamous Cell Carcinoma), SREBF1 (Sterol Regulatory Element-Binding Transcription Factor 1).

Disease	TCF19 Function	Targets/Pathways	Refs.
HCC	Promotes migration, invasion, and proliferation and adapt to metabolic stress	AKT/FOXO1, Wnt/β-catenin, c-Myc	[9,32,67,68]
NSCLC	Promotes cancer cell proliferation	Raf/MEK/ERK, *FOXO1*	[17,72]
Glioma	key oncogenic factor	*DHX32*, β-catenin	[33]
Endometrial Cancer	promotes CD8^+^ T cell exhaustion	*TRIM14*, TBK1-IRF3 and NF-κB	[14]
Osteosarcoma	Promote tumor growth and metabolic remodeling	*CDKN2A*	[35]
Breast Cancer	Promotes migration, invasion, and proliferation	miR-199a-5p, PLK1	[36,80,81]
CRC	Promote tumor proliferation and migration	WWC1	[37]
Gastric Cancer	Promotes cancer growth and invasion	-	[27]
Melanoma	Promotes cell cycles and inhibits apoptosis	-	[88]
Thyroid Cancer	Drives progression via immune modulation	-	[91]
Prostate cancer	Promotes metastatic capacity	-	[28]
HNSCC	Inhibition attenuates cell proliferation and migration	SREBF1	[92]

## 7. TCF19 Regulators

### 7.1. Indirect Inhibitors

Currently, no direct, high-affinity small-molecule inhibitor of TCF19 exists. A notable indirect strategy emerging from preclinical research involves the synergistic combination of inhibitors targeting two distinct upstream regulatory classes: Bromodomain and Extra-Terminal (BET) protein and Mitogen-activated protein kinase kinase (MEK).

**Figure 5:**
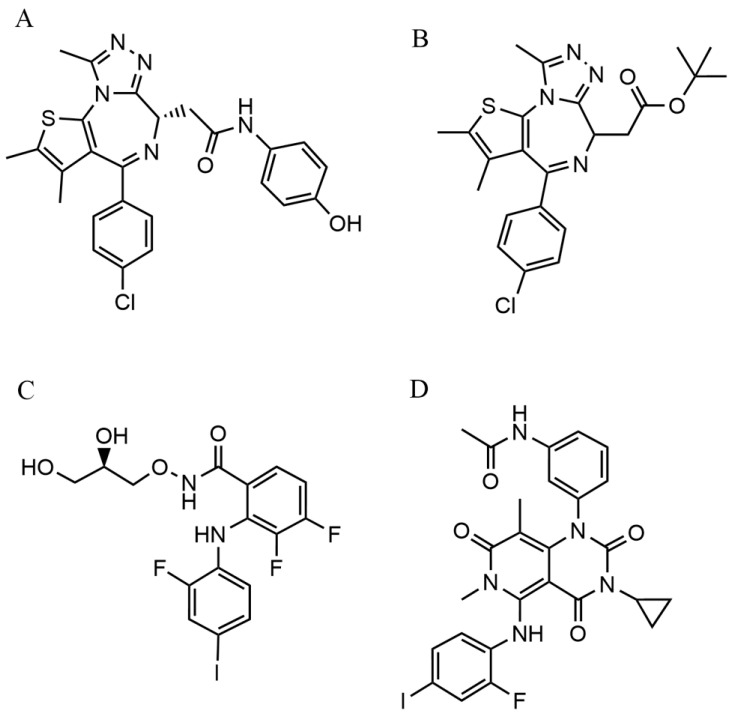
The chemical structure of TCF19 modulators. (**A**) Compound A [92]; (**B**) Compound B [92]; (**C**) Compound C [93]; (**D**) Compound D [93].

BET inhibitors, such as the clinical-stage dihydroquinazolin-2-one derivative OTX-015 (Birabresib) (Figure 5A) and the prototype thienodiazepine JQ-1 (Figure 5B), function as competitive acetyl-lysine mimetics. They bind with high affinity to the bromodomain acetyl-lysine binding pockets of proteins like BRD4, displacing them from acetylated histones and disrupting transcription complex assembly critical for TCF19 expression [93].

MEK inhibitors, exemplified by the preclinical agent mirdametinib (Figure 5C) and the FDA-approved drug trametinib (Figure 5D), are allosteric, non-ATP-competitive kinase inhibitors. They bind to a unique pocket adjacent to the ATP-binding site of MEK1/2, locking the kinase in a catalytically inactive conformation and thereby inhibiting downstream ERK signaling that drives TCF19 transcription [94].

The combination of a BET inhibitor (Figure 5A,B) and a MEK inhibitor (Figure 5C,D) exhibit potent synergy. In NRAS-mutant melanoma models, simultaneous blockade of epigenetic transcription and proximal kinase signaling converges to synergistically downregulate TCF19 mRNA and protein levels. This dual perturbation disrupts TCF19-mediated cell cycle and survival programs, inducing potent apoptosis and overcoming resistance to both MAPK-targeted therapies and immune checkpoint blockade, representing a mechanistically defined salvage strategy [88].

### 7.2. TCF19 siRNA and shRNA

Beyond indirect pharmacological approaches, RNA interference (RNAi) serves as a foundational experimental strategy for investigating TCF19 function and assessing its therapeutic potential. This approach primarily utilizes chemically synthesized small interfering RNA (siRNA) for transient knockdown and short hairpin RNA (shRNA) for stable silencing. To enhance efficacy, oligonucleotide designs often incorporate chemical modifications—such as phosphorothioate backbones and 2′-O-methyl or 2′-fluoro ribose substitutions—which improve nuclease resistance, specificity, and reduce immunogenicity [95,96]. In lung adenocarcinoma models, stable TCF19 knockdown via lentiviral shRNA delivery persistently inhibited cell proliferation, colony formation, and xenograft tumor growth, confirming its oncogenic role [72]. In MSI endometrial cancer, siRNA facilitated rapid phenotypic screening, while stable shRNA systems revealed TCF19’s role in promoting CD8^+^ T-cell exhaustion within the tumor microenvironment. Notably, in vivo delivery of TCF19-targeting shRNA via adeno-associated virus (AAV) enhanced the response to anti-PD-1 immunotherapy, underscoring the therapeutic relevance of this strategy [14]. Collectively, RNAi technologies, leveraging tailored oligonucleotide chemistry and advanced delivery vectors, provide indispensable tools for the functional dissection and therapeutic validation of TCF19 in the absence of direct small-molecule inhibitors.

## 8. Discussion

Positioned within the MHC-I region, the transcription factor TCF19 functions as a multifunctional regulator that integrates immune regulation with core cellular processes, including the cell cycle [27,30] and metabolic reprogramming [18,19,30]. This hub activity is governed by a dynamic, multi-layered regulatory network. At its core, TCF19 exerts epigenetic control through its PHD finger, which recognizes H3K4me3 to recruit chromatin remodelers, enabling precise bidirectional regulation of target genes—repressing hepatic gluconeogenic genes (G6PC, FBP1, PCK1) to maintain metabolic homeostasis [8], while activating oncogenic pathways (e.g., via DHX32) that contribute to tumorigenesis [33]. TCF19 itself is tightly regulated at transcriptional (by E2F1, VEZT) [15], post-transcriptional (by miRNAs, m^6^A methylation), and potential genetic levels. By integrating these inputs through domains like its proline-rich region [31], TCF19 orchestrates a far-reaching effector network. It acts as a core cell cycle driver at the G1/S transition by modulating cyclins, CDK inhibitors, and proliferative pathways (AKT/FOXO1, ERK, Wnt/β-catenin). It also serves as a metabolic coordinator that suppresses gluconeogenesis, promotes β-cell survival, and adjusts lipid metabolism. Additionally, it functions as an immune microenvironment modulator, epigenetically regulating MHC-I antigen presentation and driving CD8^+^ T cell exhaustion via the TCF19-TRIM14-IFN-β axis [9,21,72]. This integrative capacity underpins TCF19’s pivotal roles in cancer, metabolic disease, and immune dysfunction. Moving forward, a key immediate priority is to determine whether the FHA domain binds phospho-threonine ligands and to validate TCF19 as a druggable target via structure-guided approaches.

The transcription factor TCF19 exhibits a complex and context-dependent role across human diseases. In oncology, it functions as an oncogenic factor, with significant overexpression in malignancies such as liver and lung cancer [67,68,72] TCF19 promotes tumor progression by enhancing cancer cell proliferation, inhibiting apoptosis, facilitating metastasis, inducing CD8^+^ T-cell exhaustion in the tumor microenvironment, and reprogramming tumor metabolism [14]. Its elevated expression correlates strongly with poor prognosis and therapy resistance, underscoring its potential as a pan-cancer biomarker and therapeutic target. Conversely, in metabolic diseases like diabetes, TCF19 displays tissue-specific duality. Its genomic position within T1D susceptibility loci suggests a role in immune homeostasis and β-cell protection against autoimmunity [45,46]. In T2D, TCF19 upregulation drives compensatory β-cell proliferation and suppresses hepatic gluconeogenesis, thereby performing critical adaptive and protective functions [48,49]. Beyond these, TCF19 genetic variants are linked to CHB infection susceptibility [53,54,55]; its rare variants and m^6^A methylation regulation are associated with hearing loss; and its dysregulation contributes to inflammation in ulcerative colitis, the pathology of intervertebral disk degeneration, and gadolinium-based contrast agent toxicity [31]. Notably, in psoriasis and peeling skin disease, TCF19 exemplifies a classic “bystander gene”—occupying a key susceptibility locus without direct pathogenicity, where neighboring genes (e.g., CDSN, CCHCR1) are the primary drivers [12,13,56,58]. This paradigm offers crucial insights into disentangling causal genes in complex genetic disorders. The next step is to evaluate TCF19 expression as a clinical biomarker in well-annotated patient cohorts for both cancer prognosis and diabetes progression.

The development of direct, high-affinity small-molecule inhibitors of TCF19 remains a significant challenge. No experimental three-dimensional structure of TCF19 or its domains is currently available, which severely hampers structure-guided drug design. Current experimental modulation relies on indirect pharmacological or genetic approaches. One strategy involves suppressing TCF19 expression synergistically through inhibitors of upstream regulatory pathways, such as BET and MEK inhibitors, which has shown promise in overcoming therapy resistance in models like NRAS-mutant melanoma [93,94]. Genetic knockdown via RNA interference (siRNA/shRNA) remains the principal tool for functional validation, with studies consistently demonstrating that TCF19 ablation impedes tumor proliferation, metastasis, and immune evasion [14,72]. The primary barrier to direct inhibitor development is a lack of high-resolution structural data, particularly co-crystal structures of its PHD domain bound to its cognate H3K4me3 mark or interacting partners, which hampers structure-guided drug design. Moreover, as a transcription factor that functions primarily through protein-protein and protein-histone interactions, TCF19 lacks a classic small-molecule binding pocket, further complicating direct inhibitor development. Thus, a short-term milestone should be the determination of the PHD finger-H3K4me3 co-crystal structure, enabling virtual screening for small-molecule disruptors. Furthermore, a major limitation of current studies is the heavy reliance on cancer cell lines and xenograft models; few have validated findings in immunocompetent, genetically engineered mouse models or patient-derived organoids, which are needed to assess therapeutic relevance and off-target effects.

Advancing TCF19 from a critical biological hub to a viable therapeutic target requires addressing fundamental mechanistic questions and translational hurdles. Key priorities include elucidating the phosphopeptide recognition specificity and signaling role of its poorly characterized FHA domain; resolving the tissue-specificity paradox of its dual pro-proliferative functions in cancer cells versus pancreatic β-cells; and detailing the spatiotemporal regulation of the TCF19-TRIM14-IFN-β axis that drives CD8^+^ T-cell exhaustion. Concurrently, rigorous validation of TCF19 as a clinical biomarker across diverse cancers and metabolic diseases is essential for translational relevance.

A coordinated, multi-pronged strategy is needed to overcome these challenges. Structurally, determining the three-dimensional architecture of full-length TCF19 and its key domain complexes is foundational for revealing druggable sites. Therapeutically, the field must explore innovative modalities beyond traditional inhibitors, including proteolysis-targeting chimeras (PROTACs) for TCF19 degradation, allosteric modulators, and optimized indirect combination strategies—such as co-targeting the TCF19-TRIM14 axis with PD-1 blockade. Near-term viable avenues include synergistic applications of existing pathway inhibitors (e.g., BET and MEK inhibitors). Central to translation will be overcoming potential tissue-specific toxicity and compensatory network effects. Ultimately, success will depend on integrating structural biology, innovative chemical approaches, and a system-level understanding of TCF19’s context-dependent functions to accelerate the development of diagnostics and therapeutics for cancer, metabolic, and inflammatory diseases.

## 9. Conclusions

In summary, TCF19 is a central transcriptional hub that integrates cell cycle, metabolism, and immunity via its PHD finger-mediated epigenetic control. Its context-dependent duality—pro-oncogenic in cancers yet protective in metabolic diseases—positions it as both a therapeutic target and a biomarker. The lack of high-resolution structures for its PHD and FHA domains remains the main bottleneck for drug design. Overcoming this, alongside validation in immunocompetent models and patient cohorts, is essential. Future priorities include determining domain co-crystal structures, exploring PROTACs, and develop small-molecule inhibitors that directly bind to translate TCF19 biology into diagnostics and therapeutics for cancer, metabolic, and inflammatory diseases.

## Data Availability

The authors confirm the availability of all data upon reasonable request.
